# Exploring the Motivators and Barriers of Older Adults Participating in an Interactive Exergame Intervention

**DOI:** 10.1093/geroni/igaa057.1293

**Published:** 2020-12-16

**Authors:** Breanna Crane, Brittany Drazich, Kyle Moored, Michelle Carlson

**Affiliations:** 1 Johns Hopkins Bloomberg School of Public Health, Baltimore, Maryland, United States; 2 Johns Hopkins School of Nursing, Baltimore, Maryland, United States

## Abstract

Cognitive and physical activity are important to maintaining daily functioning in older adults. While bidirectional associations between cognitive and physical activity magnify with increasing age, elucidating shared benefits has been difficult as few interventions explicitly train on cognition and mobility simultaneously. We conducted focus groups among 14 older adults residing in an independent-living center who participated in an interactive video game study called Bandit the Dolphin, where participants simultaneously incorporated cognitive exercise and physical activity while navigating within a complex spatial environment to help Bandit jump, eat fish, and stun sharks. Using ‘sneaky exercise’ tactics, participants utilized upper extremities in conjunction with slight lower extremity movement to move Bandit within a 3-D oceanic environment. We conducted 3 semi-structured focus groups and analyzed the data using the “Sort and Sift, Think and Shift” method to assess general likes and dislikes as well as the primary motivators, barriers, and reasons for remaining in the study. Participants enjoyed the immersive nature, challenge, and “fun factor” of the game. Primary motivators for joining were generativity/helping others, self-improvement, from peer referrals, and because the study looked interesting. Key barriers reported in the study were exhaustion from standing, learning how to play in 3-D space, and frustration from lack of level advancement. Reasons for retention were due to the game being fun, a sense of duty, and fulfilling commitments. This information will guide ongoing research efforts to design interactive video game interventions that are enjoyable for older adults and maintain high retention rates.

